# Regenerative Capacities of Chitosan-Nanoselenium Conduit on Transected Sciatic Nerve in Diabetic Rats: An Animal Model Study

**DOI:** 10.29252/beat-080103

**Published:** 2020-01

**Authors:** Darab Faraji, Mohsen Ebrahimi, Babak Paknezhad, Zahra Hami, Alireza Jahandideh

**Affiliations:** 1 *Faculty of Medicine, AJA University of Medical Sciences, Tehran, Iran*; 2 *Department of Toxicology and Pharmacology, Faculty of Medicine, AJA University of Medical Sciences, Tehran, Iran*; 3 *Department of Clinical Science, Faculty of Specialized Veterinary Sciences, Science and Research Branch, Islamic Azad University, Tehran, Iran*

**Keywords:** Peripheral nerve repair, Sciatic, Chitosan-nanoselenium conduit, Local

## Abstract

**Objective:**

To assess regenerative capacities of chitosan-nanoselenium conduit on transected sciatic nerve in diabetic rats.

**Methods:**

A 10-mm sciatic nerve defect was bridged using a chitosan-nanoselenium conduit filled with phosphate buffered saline. In chitosan group, the chitosan conduit was filled with phosphate buffered saline solution. In sham-operated group, sciatic nerve was exposed and closed. In transected group, right sciatic nerve was transected and nerve cut ends were fixed in the adjacent muscle. The regenerated fibers were studied within 12 weeks after surgery.

**Results:**

The behavioral and functional and electrophysiological tests confirmed faster recovery of the regenerated axons in chitosan-nanoselenium conduit group compared to chitosan group (*p=*0.001). The mean ratios of gastrocnemius muscles weight were measured. There was statistically significant difference between the muscle weight ratios of chitosan-nanoselenium conduit and chitosan groups (*p=*0.001). Morphometric indices of regenerated fibers showed number and diameter of the myelinated fibers were significantly higher in chitosan-nanoselenium conduit group than in chitosan group.

**Conclusion:**

chitosan-nanoselenium conduit resulted in acceleration of functional recovery and quantitative morphometric indices of sciatic nerve.

## Introduction

Lots of patients suffered from traumatic peripheral nerve injuries every year [1]. A considerable proportion of these patents were young people who constituted the main labor force of the society [2]. The nerve injuries caused the extremities dysfunction and affected their work and living abilities, which brought the society with a huge burden. The functional recovery of peripheral nerve injuries is associated with many factors, including the time interval between the injury and repair surgery [3]. 

However, some patients could not be treated with the nerve injuries such as the presence of multiple fractures requiring aggressive and expeditious management in preference to the nerve injuries [3]. The delayed nerve injuries treatment resulted in poor outcome of the extremity sensory and motor functions [4]. Studies in the control of diabetes mellitus and diabetic neuropathy [5] have renewed the interest in the rate and quality of nerve regeneration in this chronic disease. Although measurable improvements may follow better control of blood sugar and administration of aldose reductase inhibitors, complete recovery is dependent on the regeneration of damaged axons and the reestablishment of fully functional connection with their targets [6].

Several nerve guidance conduits (NGCs) and nerve protectant wraps are approved by the US Food and Drug Administration (FDA) for clinical use in peripheral nerve repair. These devices cover a wide range of natural and synthetic materials, which may or may not be resorbable [7]. Biodegradable nerve guides as a temporary scaffold are better than non-degradable biomaterials because the latter remain *in situ* as a foreign body and ultimately result in limiting recovery of nerve function [8]. 

Nevertheless, the resistance to biodegradation can be a cause of chronic nerve compression in the long run and a second surgery may therefore be required for its removal. Beneficial effects of chitosan as a conduit in promoting nerve regeneration have already been documented and it seems chitosan as a natural polymer has excellent properties including biocompatibility, biodegradability, non-toxicity and adsorption properties, and might be a suitable functional material for peripheral nerve regeneration [9, 10].

Selenium is one of the essential trace elements for humans. The bioavailability of selenium is related to its different chemical species. Recently, elemental selenium nanoparticles are attracting more and more attention due to their excellent high biological activity and lower toxicity [11]. Elemental selenium nanoparticles in liquid phase can be used as the materials for medical purposes [12]. For these applications, it is important to have good stability of elemental selenium nanoparticles in liquid phase [13]. 

One of the effective methods for stability of nanoparticles in liquid phase is to add modifiers. Others used the chitosan as modifiers for the fabrication of elemental selenium nanoparticles [13]. Because of absence of available data on beneficial effects of nanoselenium on peripheral nerve regeneration, the present animal model study was conducted to assess regenerative capacities of chitosan-nanoselenium conduit on transected sciatic nerve in diabetic rats.

## Materials and Methods

*Study design*

Sixty male White Wistar rats weighing approximately 280 g were randomly divided into four experimental groups (n=15) including transected control group, sham-operated group, a chitosan conduit group and a chitosan-nanoselenium conduit group. Each group was again subdivided into three subgroups of five animals each and surveyed within12 weeks. Two weeks before and during the entire experiments, the animals were housed in individual plastic cages with an ambient temperature of 23±3º C, stable air humidity, and a natural light/dark cycle. The rats had free access to standard rodent laboratory food and tap water. All protocols were based working with laboratory animals of Iran Veterinary Organization.

*Preparation of chitosan- nano selenium biodegradable matrix*

Water-soluble chitosan solution was prepared using a method described by others [14]. Briefly, Medium molecular weight crab shell chitosan was dissolved (~400 kDa, 85% deacetylated, Sigma- Aldrich St. Louis, MO, USA) into an aqueous solution (1% v/v) of glacial acetic acid (Merck, Germany) to a concentration of 2% (w/v), while stirring on a magnetic stirrer-hot plate. The solution was stirred with low heat (50˚C) for 3 hours. The resultant chitosan solution was filtered through Whatman filter paper after vacuum filtration to remove any un-dissolved particles. 

For the preparation of elemental selenium nanoparticle sol, 1.5 mL 0.227 mol/L Vc was mixed with 1.0 mL 2.40 mol/L acetum, then the appropriate amounts of 5.36 mmol/L Se (IV) solution was added into the mixtures, the mixed solution was diluted to 10 mL. For the preparation of selenium nanoparticle-chitosan solution, appropriate amounts of chitosan solution were mixed with 1.5 mL 0.227 mol/L Vc and 1.0 mL 2.40 mol/L acetum, respectively. 

**Fig. 1:**
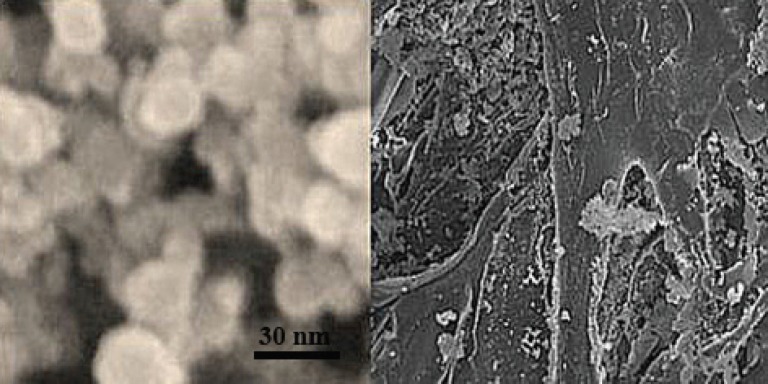
(**A**) TEM micrograph of selenmium nano particles confirmed that nanoselenium nanoparticles were observed almost sphere like in morphology and approximately 30 nm in diameter. (**B**) SEM micrograph of chitosan-nanoselenium conduit

The appropriate amounts of 5.36 mmol/L Se (IV) solution was added into the mixtures, then the mixed solution was all diluted to 10 mL. Philips diffractometer was used to obtain X-ray diffraction pattern. X-ray diffraction (XRD) patterns were acquired from 2θ=10° to 80° using Cu Kα1 radiation. Transmission electron microscope (Philips ES 30 KW0) was used to determine the size of nanoparticles (Figure 1).

*Surgery*

Animals were anesthetized by intraperitoneal administration of ketamine-xylazine (ketamine hydrochloride 5%, 90 mg/kg and xylazine hydrochloride 2%, 5 mg/kg). The procedures were carried out based on the guidelines of the Ethics Committee of the International Association for the Study of pain [15]. The University Research Council approved all experiments. 

Following surgical preparation in the sham-operated group, the right sciatic nerve was exposed through a gluteal muscle incision and after careful homeostasis the muscle was sutured with resorbable 4/0 sutures, and the skin was closed with 3/0 nylon. 

In transected control group, the right sciatic nerve was transected proximal to the tibio-peroneal bifurcation, where a 7 mm segment was excised, leaving a 10 mm gap due to retraction of nerve ends. Proximal and distal stumps were fixed in the adjacent muscle with 10/0 nylon epineurial suture. No graft was interposed between the stumps. In the chitosan group, the right sciatic nerve was exposed through a gluteal muscle incision and transected proximal to the tibio-peroneal bifurcation where a 7 mm segment was excised, leaving a gap about 10 mm due to retraction of nerve ends. 

Proximal and distal stumps were each inserted 2 mm into a chitosan conduit and two 10/0 nylon sutures were placed at each end of the cuff to fix the tube in place and to leave a 10-mm gap between the stumps. The conduit was filled with 20 µL phosphate buffered saline. In the chitosan-nanoselenium conduit group, the chitosan-nanoselenium conduit was filled with phosphate buffered saline. The sterile vaseline was used to seal the ends of the tubes to avoid leakage. Currently acceptable technique of euthanasia in neurosciences include perfusion fixation. The animals were similarly anesthetized and euthanized with transcardial perfusion of a fixative containing 2% paraformaldehyde and 1% glutaraldehyde buffer (pH=7.4) 4, 8 and 12 weeks after surgery. 

*Behavioral test*

Functional recovery of the nerve was assessed using the Basso, Beattie, and Bresnahan (BBB) locomotor rating scale for rat hind limb motor function [16]. Although BBB was widely used to assess functional recovery in spinal cord injured animals; however, it has been demonstrated that it could be most useful in assessment of never repair processes in peripheral nerve injuries [17]. The testing was performed in a serene environment. The animals were observed and assessed within a course of a 4-minute exposure to an open area of a mental circular enclosure. BBB scores were recorded once before surgery in order to establish a baseline control and again weekly thereafter to assess functional recovery during 12 weeks. 

*Functional assessment of re-innervation-sciatic functional index (SFI)*

Walking track analysis was performed 4, 8 and 12 weeks after surgery based on others [18]. The lengths of the third toe to its heel (PL), the first to the fifth toe (TS), and the second toe to the fourth toe (IT) were measured on the experimental side (E) and the contralateral normal side (N) in each rat. The SFI in each animal was calculated by the following formula as SFI=- 38.3×(EPL-NPL)/NPL+109.5×(ETS-NTS)/NTS+13.3×(EIT-NIT)/NIT-8.8.

*Electrophysiological studies*

After 12 weeks following the track test, all animals were subjected to electrophysiological studies using Nacro bio system (320-3760 A trace 80, USA). Under general anesthesia, the left sciatic nerve was re-exposed by incision of the skin at the previous surgical site. Single electrical pulses at supra-maximal intensity were delivered via bipolar electrodes placed in turn at the proximal and distal trunk of the regenerated nerve. Electromyography (EMG) was recorded by inserting an electrode into the belly of gastrocnemius muscle. 

**Fig. 2:**
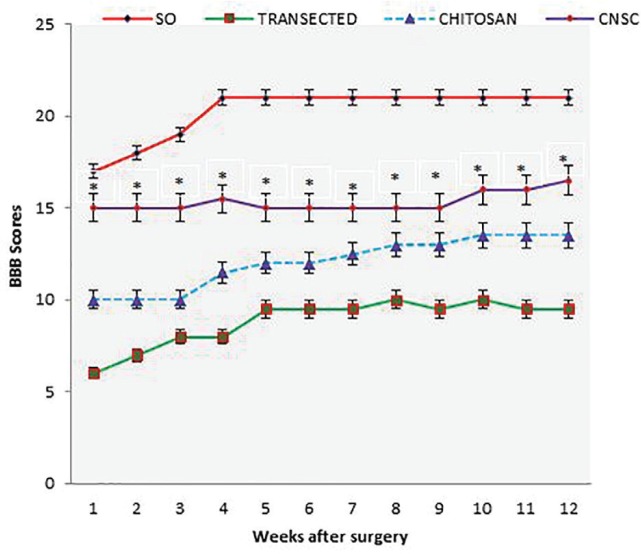
BBB score for all experimental groups. Chitosan-nanoselenium conduit grafting gave better scores than in chitosan group. Standard error at each data point is shown with bars

**Fig. 3:**
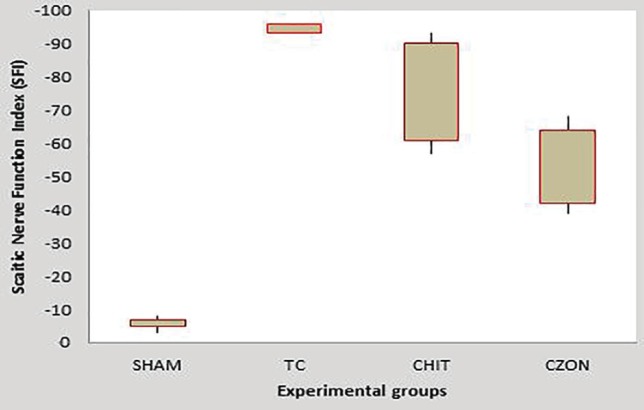
Box-and-whisker plots of sciatic nerve function index values in each experimental group during the study period. Chitosan-nanoselenium conduit grafting gave better results in functional recovery of the sciatic nerve than in chitosan group

**Fig. 4:**
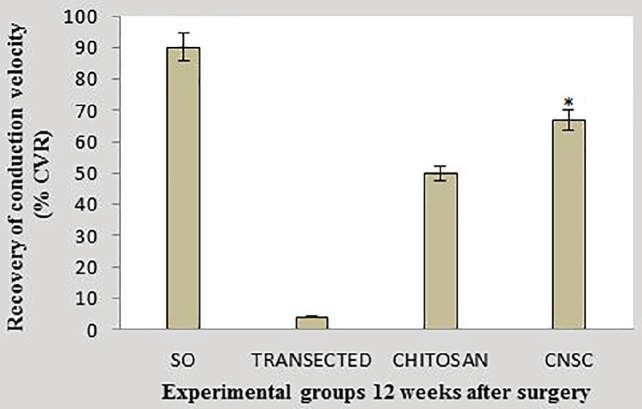
Percentage recovery of conduction velocity in experimental groups. Data are presented as mean±SD. *P=0.001 *vs* chitosan group

**Fig. 5:**
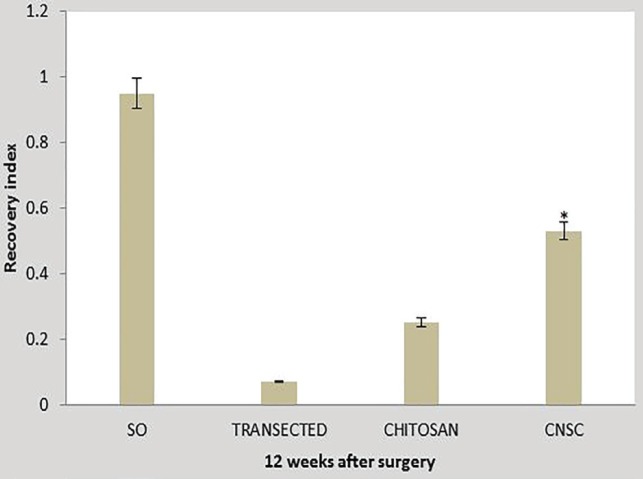
Recovery index in experimental groups. Data are presented as mean±SD. *P=0.001 *vs* chitosan group

**Fig. 6:**
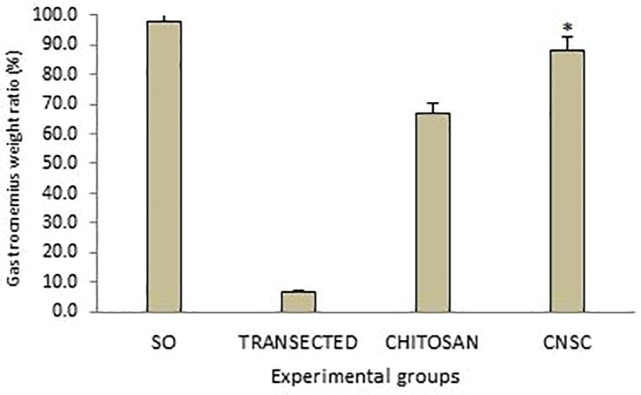
Gastrocnemius muscle weight measurement. The gastrocnemius muscles of both sides (operated right and unoperated right) were excised and weighed in the experimental groups at 12 weeks after surgery. Data are presented as mean±SD. *P=0.001 vs chitosan group

**Fig. 7.**
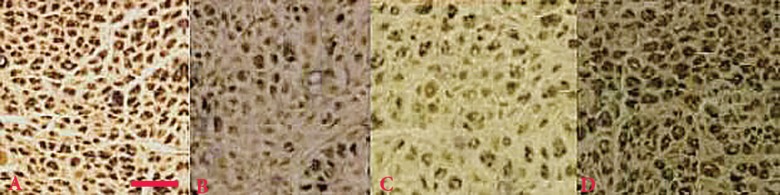
Osmium staining of distal nerve stumps of the regenerated nerves 12 weeks after surgery from middle cable (**A**) sham-operated, (**B**) transected, (**C**) chitosan and (**D**) chitosan-nanoselenium conduit groups. Scale bar: 30 μm

**Table 1:** Biomechanical analyses of sciatic nerve in each of the experimental groups: Values are given as mean±SD

**Groups**	**Tensile strength** **(MPa) **	**Maximum pull force ** **(N)**	**Toughness** **(N/mm)**	**Ultimate strain**
**Sham-operated**	7.30±1.20	5.40±0.40	1.25±0.55	0.53±0.09
**Transected**	2.33±0.30	1.40±0.15	0.23±0.08	0.13±0.07
**Chitosan**	3.10±0.13	3.17±0.30	0.43±0.20	0.23±0.04
**Chitosan-nanoselenium conduit**	4.37±0.15^a^	4.34±0.24^ a^	0.78±0.10^ a^	0.38±0.05^ a^

^a^The mean difference is significant at the 0.05 level *vs.* chitosan group.

**Table 2:** Morphometric analyses of sciatic nerve in each of the experimental groups: Values are given as mean±SD

**Groups**	**Myelin sheath thickness (µm)**	**Axon diameter** **(µm) **	**Axon counts** **fb/mm** ^2^
**Sham-operated**	2.65±0.05	11.35±0.15	29068±2104
**Transected**	1.05±0.03	3.35±0.13	4103±2103
**Chitosan**	1.04 ±0.03	3.70±0.12	21347±2391
**Chitosan-nanoselenium conduit**	1.04±0.07	6.44±0.15^a^	23172±2102^a^

^a^The mean difference is significant at the 0.05 level *vs.* chitosan group.

The latency and the amplitude of EMG were obtained. Also, the difference in latency of EMG was measured, and the distance between the proximal and distal sites of stimulation was measured to calculate the conduction velocity across the regenerated nerve. On the contralateral, right intact side of each animal, similar measurements were made for the determination of conduction velocity. The conduction velocity of the bridged nerve was expressed as a percentage of that on the intact side of each animal to cancel off variations between animals (% CVR). The recovery index of EMG amplitude in all groups was calculated based on Suzuki *et al.* using the following formula: Recovery index=Peak amplitude of the operated side/Peak amplitude of the intact side [19].

*Biomechanical tests*

The regenerated nerves were harvested and placed in a normal saline bath at room temperature. The samples were then fixed between frozen fixtures in a mechanical apparatus. The TA.XTPlus Texture Analyzer mechanical test device was used for the assessment (Stable Micro Systems, Surrey GU7 1YL, UK). After 5 minutes, the frozen fixtures were tightened to ensure that no slippage occurred during testing. The initial length was set to 10 mm. Each sample was stretched at a constant rate of 1 mm/min. The load and displacement were sampled 5 times per second. Each sample was stretched to complete tensile failure. Samples were kept wet moist during testing using a drop of normal saline solution to the nerve segments.

*Muscle mass*

Recovery assessment was also indexed using the weight ratio of the gastrocnemius muscles, 12 weeks after surgery. Immediately after sacrificing of animals, gastrocnemius muscles were dissected and harvested carefully from intact and injured sides and weighed while still wet, using an electronic balance. All measurements were made by two independent observers unaware of the analyzed group.

*Osmium tetroxide staining and quantitative morphometric studies*

Four and 8 weeks after the 2^nd^ surgery, 6 rats in each group were deeply anesthetized with 10% chloral hydrate (3.5 ml/kg weight intraperitoneally) and perfused through the left ventricle. Totally, 250 ml of warm saline flush was followed by 250 ml of ice-cold 4% paraformaldehyde in 0.1M, pH 7.4 phosphate buffer. After perfusion, the entire nerve, including the 1 cm from the suture proximally and 2 cm distally, was removed from each rat. One centimeter nerve segment proximal to the bifurcation was harvested and fixed in 4% paraformaldehyde in 0.1M phosphate buffer for 24 hours at 4℃.

It was later stained in 1% osmium tetroxide for 12 hours and then dehydrated through a graded series of ethanols, and specimens were then immersed in xylene, embedded in paraffin, and sliced into 2-μm cross-sections. Images were acquired under light microscopy (Olympus, Tokyo, Japan), from which the total number of myelinated axons and myelin thicknesses and diameters were evaluated. Morphometric measurements were performed using Image-pro Plus 5.0 software (Media Cybernetics). The shortest lengths of the outer and inner margins of the myelin sheath were measured to determine the fiber diameter and axon diameter. Myelin thickness was calculated after the fiber and axon diameters were determined.

*Statistical Analysis*

Experimental results were expressed as means±SD. Statistical analyses were performed using PASW 18.0 (SPSS Inc., Chicago, IL, USA). Model assumptions were evaluated by examining the residual plot. Results were analyzed using a factorial ANOVA with two between-subject’s factors. Bonferroni test for pairwise comparisons was used to determine the effect of time and treatments. The differences were considered significant when *p*< 0.05.

## Results

*BBB recovery*

In order to assess hind limb recovery, the open field locomotor was used. Figure 2 shows BBB scores compared to the baseline. All groups, except for sham-operated group, showed the greatest degree of functional deficit one week after surgery. The chitosan-nanoselenium conduit group showed significant improvement in locomotion of the operated limb compared to the chitosan group during the study period (*p*= 0.001). 

*Recovery of sciatic nerve function and reinnervation-SFI outcome*

Figure 3 shows SFI values in experimental groups. Prior to surgery, SFI values in all groups were near zero. After the nerve transection, the mean SFI decreased to -100 due to the complete loss of sciatic nerve function in all animals. The statistical analyses revealed that the recovery of nerve function was significantly faster in chitosan-nanoselenium conduit group than in chitosan group (*p*=0.001).

*Findings of electrophysiological studies*

Figure 4 and 5 show nerve conduction velocity (NCV) along regenerated sciatic nerves in experimental groups. NCV in chitosan-nanoselenium conduit group was significantly higher than that in chitosan group (*p*=0. 001). 

*Biomechanical measurements*

F_max_ of nerve samples in experimental groups were shown in Table 1. F_max _in chitosan-nanoselenium conduit group was significantly higher than that in chitosan group (*p*=0. 001). Tensile strength, the amount of force per unit of initial cross-sectional area at tensile failure, was measured based on F_max _and nerve cross sectional area. 12-week assessment revealed tensile strength of regenerated nerves treated with ibuprofen was higher than those in chitosan group (*p*=0. 001). Ultimate strain, the amount of elongation divided by the initial specimen length achieved at the point of tensile failure, in chitosan-nanoselenium conduit group was significantly higher than that in chitosan group (*p*=0. 001). Toughness, reflecting the properties of anti-deformation and anti-fracture of the nerve, was determined by the nerve itself and could reflect “looseness” or “toughness” of the nerve. Toughness in chitosan-nanoselenium conduit group was significantly higher than that in chitosan group (*p*=0. 001).

*Muscle mass measurement*

Gastrocnemius muscles weight of injured and uninjured sides were measured in each group. There was statistically significant difference between percentage of the mean muscle weight ratios of chitosan-nanoselenium conduit and chitosan groups (*p=*0.001). The findings showed that in chitosan-nanoselenium conduit group, the muscle weight ratio was bigger than in chitosan group (Figure 6). 

*Morphometric findings*

Table 2 shows quantitative morphometric analyses of regenerated nerves for each of the experimental groups. Four weeks after surgery, chitosan-nanoselenium conduit group presented significantly greater nerve fiber, axon diameter and myelin sheath thickness compared to chitosan group (*p=*0.001). Although chitosan group presented regeneration patterns, the morphometric indices in chitosan-nanoselenium conduit group, both after 8 and 12 weeks were better than in chitosan (Figure 7). 

Using Factorial ANOVA analysis with two between-subject’s factors (Group×time); in the chitosan-nanoselenium conduit group, the number of nerve fibers and myelin thickness did not show significant difference between 8 and 12 weeks intervals (*p=*0.001). Mean thickness of myelin sheath from week 8 onward did not show significant difference between chitosan-nanoselenium conduit group and chitosan group (*p=*0.001).

## Discussion

Peripheral nerve injuries accounts for a considerable part of traumatic injuries around the world, especially in vehicle accidents and fall from a height. Such patients usually have other coalescent sufferings which must be treated in preference. So the nerve repair surgery would be postponed till the major injuries were controlled. In such condition, the neuronal body cannot receive the neurotrophic factors from the end organ in terms of retrograde axonal transport and die in consequences [20]. 

The nerve fibers degenerate and the muscle atrophies [21]. Poor outcome from peripheral nerve injury is especially evident when repair is delayed [22]. In order to improve the poor functional outcome of delayed nerve repair, some studies proposed effective methods to promote axonal regeneration. Nerve conduction measurement is a direct evidence for the study of nerve transmission [23]. The conduction velocity depends on the diameter of axons and the thickness of myelin sheath [24]. 

The results of the present study showed significantly different conduction velocity between the ibuprofen treated animals and eggsell membrane (ESM) bridged regenerated sciatic nerves, therefore, the ESM conduit in combination with ibuprofen could be assumed as a safe technique with no nerve conduction interference. The strongest connective tissue layers in peripheral nerves are the perineurium and, to a lesser extent, the epineurium. Changes in the epineurium and perineurium extracellular matrix composition are likely to have significant effects on the biomechanical properties of acellular nerve [25]. 

The connective tissue from the epineurium forms a layer of fiber membrane at the 3^rd^ day postoperatively and then forms collagen at the 8^th^ day. The key point influencing functional recovery is the number of axons throughout the suture that enhances the anti-tension capacity of the nerve [26]. Application of ibuprofen to regenerated nerve in the present study resulted in the enhanced biomechanical indices that were in agreement with functional and morphometric findings. 

It is known from previous studies that regeneration process in rats would not have been completed by 12 weeks, a phenomenon which has been reported in a variety of experimental models [27]. Quantitatively, our results are consistent with these findings. However, a 12-week experimental period is sufficient for evaluation of regeneration process because in rats functional recovery after repair of a transected peripheral nerve occurs during this timeline [28].

The results of the present study showed that chitosan-nanoselenium conduit accelerated sciatic nerve functional recovery in diabetic rats. Nerve conduction measurement is a direct evidence for the study of nerve transmission [29]. The conduction velocity is dependent on the diameter of axons and the thickness of myelin sheath [24]. Our findings demonstrated that there was significantly different conduction velocity between cell treated animals and vein graft bridged regenerated sciatic nerves. Therefore, the chitosan conduit in combination with nanoselenium could be assumed as a safe nerve guide with no interference in nerve conduction.

Arrival of sprouts from the proximal stump at the distal nerve stump does not necessarily imply recovery of nerve function [28]. BBB is widely used to assess functional recovery in spinal cord injured animals, however, it has been demonstrated that it could be most useful in assessment of never repair processes in peripheral nerve injuries [30]. Results of the present study showed that chitosan-nanoselenium conduit treated animals were improved in locomotion of the operated limb compared to the control group during the study period.

Walking track analysis and static sciatic index has frequently been used to reliably determine functional recovery following nerve repair in rat models [31, 32]. It is a coordinated activity involving sensory input, motor response and cortical integration [28]. Recording wet muscle weight is a previously utilized alternative for motor target organ reinnervation [33-35]. As the posterior tibial branch of the sciatic nerve regenerates into the gastrocnemius muscle, it will regain its mass proportional to the amount of axonal reinnervation [36, 37]. 

At the end of the present study, chitosan-nanoselenium conduit treated group showed significantly greater ratios of the mean gastrocnemius muscle weight in comparison to chitosan group indicating indirect evidence of successful end organ reinnervation. Quantitative morphometrical indices of regenerated nerve fibers showed significant difference between chitosan-nanoselenium conduit group and chitosan groups. Regarding better functional and morphometric indices in chitosan-nanoselenium conduit group versus chitosan group at the end of the study period, it could be stated that cell therapy, both accelerated and improved the process of nerve regeneration. 

Despite much effort to introduce ideal therapeutic drugs for diabetic neuropathy, aldose reductase inhibitors have been shown to be the most established compounds among potent drugs. However, although experimental data on aldose reductase inhibitor have been very promising, their clinical efficacy seems limited even for mild degrees of diabetic neuropathy. Neurotrophic factors appear less effective than such conventional drugs; no extensive trials have shown their efficacy and a considerable number of adverse effects are also problematic [38]. 

Ongoing research in the field of electrically active nanomaterials includes the fabrication of composite materials with nanoscale, nanoselenium particles embedded into a polymer matrix. Nanoselenium, when mechanically deformed through ultrasound, for example, can theoretically provide an electrical stimulus, a known stimulatory cue for neural tissue regeneration. The combination of nanoscale surface dimensions and electrical activity may provide an enhanced neural tissue regeneration environment; such multifaceted nanotechnology approaches deserve further attention in the neural tissue regeneration field [39].

Even though our study showed the neuroregenerative action of chitosan-nanoselenium conduit in peripheral nerve injuries, data regarding the molecular mechanisms leading to the neuroprotective action remain to be investigated in depth. We have not given molecular evidence for neuroprotective action of chitosan-nanoselenium conduit. This may be considered as a limitation to our study. 

In conclusion, this study demonstrated that chitosan-nanoselenium conduit improved functional recovery of transected sciatic nerve in rat. Supported by previous findings, the results from our present study would imply that the final outcome of both motor and sensory regeneration and reinnervation following repair of a peripheral nerve by chitosan-nanoselenium conduit may be of clinical benefit. There are reasonable grounds to believe that this approach could deliver a superior quality of reinnervation in a shorter period of time, compared to repair without nanoparticle treatment.
